# Emetics or Brisk Cathartics in Strumous Ophthalmia

**Published:** 1884-02

**Authors:** R. F. Henry

**Affiliations:** Princeville, Illinois


### ARTICLE III.

### EMETICS, OR BRISK CATHARTICS IN STRUMOUS OPHTHALMIA.

By R. F. HENRY, M.D., Princeville, Illinois.

About ten years since, I treated a case of strumous ophthalmia or phlyctenular keratitis,for several weeks without giving any permanent relief to the suffering child. I consulted the best authorities I could find and called a reputable specialist who visited and prescribed for the case. Various local applications were made to the eye; mild cathartics were administered; tonics and alteratives, nourishing food, etc., were used, but the case grew worse and worse until I became disheartened and alarmed. I then administered an emetic of ipecacuanha, the good effects of which were manifested in a few hours afterwards.

The photophobia, which was intense, was relieved so that the child could open its eyes in the light. Improvement commenced in the swollen lids and pustular prominences on the cornea, all the bad symptoms rapidly disappeared without any further local treatment, and with the use of tonic doses of quinine and nutritious diet the patient was soon restored to good health, and has never had any return of the disease.

In all the patients I have treated since the above, I have given the emetic when I first visited them, whether it was at the commencement of the disease or at whatever stage; or whether they had been submitted to previous treatment. I have not found it necessary in any of these cases to use any local treatment to the eye, except rest to the eyelids, exclusion from light, and repeated fomentations with warm water.

I would not hesitate to repeat the emetic if necessary, but all the cases which I have treated since I learned the practice have recovered so rapidly that no repetition was called for. In one case I administered a cathartic of rhubarb and calomel after the emetic had operated, but I am not sure that the case would not have recovered without it. If any one should hesitate to give the emetic on account of any great fear as to its depressing effects I can assure him that in every case in which I have used it, quite the contrary has been the effect, the system rallied in place of being more depressed under the action of the remedy. For suggesting the above treatment I am indebted to the *London Lancet*, vol. 1, No. 2, 1859, in which was published a lecture on strumous ophthalmia delivered in Middlesex Hospital by Mitchell Henry, Esq., F.R.C.S., Surgeon to the Hospital and to the North London Ophthalmic Infirmary. With regard to the name of the disease the lecturer said "it is not happily chosen, for the ocular affection is by no means peculiar to the scrofulous, the worst cases being often free from all glandular affections, strumous abscess, or tubercular deposit; it is true that you will often find it associated with all the hideous features of scrofula, but in many instances the disease is merely the local evidence of constitutional disorder, where there is is no pretense for imputing the strumous diathesis to the sufferer. Why it is that in some young children any irritation of the primæ vicæ is attended with sore eyes, and in others with diarrhœa or stomach cough, or a genuine flux of the bronchial mucous membrane, or not infrequently with convulsions, are secrets belonging to the mysteries of vital peculiarities which are to us at present inexplicable. The fact should, however, at once suggest to us a caution not to treat any of these affections as if they were mere local ones, lest by injudicious applications we convert what are mere symptoms of bodily disorder into genuine obstinate diseases."

"Regarding the disorder as an indication of an acid and foul condition of the primæ viæ—an outward sign of a constitutional disorder—you must proceed to treat it as you would any such complaint in another part of the body." The author then says that "the first measure must be the administration of an active purgative to clear away that thick and peccant mucus which always occupies the intestines of delicate, ill-nourished children. For this purpose there is nothing like calomel, for, as a purgative, it is often invaluable. It acts strengthening on the delicate glandular structure of the intestines, just as, continued for some time, it acts at last on the salivary glands of the mouth, and produces from them a copious pouring out of saliva. The best combination is calomel, rhubarb and scammony." On children, the lecturer said, "An emetic will often act like a charm, and, for the moment, subdue at once the photophobia and the intolerable itching; but in adults such remedies are not so readily submitted to, and you must be content with purgatives." I cannot, from my limited experience, speak with so much assurance as to the good effects of active cathartics, but the author above quoted said in the same lecture that it was "surprising to see how a child would sometimes pick up and gain in a few days some pounds of weight after the operation of a brisk cathartic. The mucus is cleared away, and the lacteals left free to absorb the chyle with which, for the first time, they come in contact. Never, then, forget to administer an occasional purgative to both old and young when these disorders of mal-assimilation are concerned." As to the results of this treatment which had been practiced by him for six years and by several of his colleagues at the eye infirmary for ten years, he said, "They have been most gratifying; glandular lids, chronic inflammation of the tarsal margins, ingrowing eye-lashes, are almost unknown to us except in cases that have formerly been submitted to the stimulating plan."

In setting so much importance on this remedial measure I do not wish to be understood to undervalue the other means of restoring health, such as diet and regimen, aids in digestion, if necessary, alteratives and tonics, and common-sense local applications. I believe they are all good in their place, and some are indispensable, but I am convinced that they will do more good after the operation of an emetic than they will before.

