# Correction

**Published:** 1884-04

**Authors:** 


CORRECTION.—In the February issue of this journal, in the original communication of Dr. R. F. Henry, Article III., page 130, fifth line from the top, the word "strengthening" is printed in place of "straightway."

